# Distinct Metabolome Changes during Seed Germination of Lettuce (*Lactuca sativa* L.) in Response to Thermal Stress as Revealed by Untargeted Metabolomics Analysis

**DOI:** 10.3390/ijms21041481

**Published:** 2020-02-21

**Authors:** Shiwei Wei, Xiao Yang, Guotao Huo, Guojun Ge, Hongyan Liu, Lijun Luo, Jinguo Hu, Danfeng Huang, Ping Long

**Affiliations:** 1 Shanghai Agrobiological Gene Center, Shanghai 201106, China; wsw@sagc.org.cn (S.W.); hgt@sagc.org.cn (G.H.); lhy@sagc.org.cn (H.L.); lijun@sagc.org.cn (L.L.); 2 Institute of Urban Agriculture, Chinese Academy of Agricultural Sciences, Chengdu National Agricultural Science and Technology Center, Chengdu 610213, China; yangxiao@caas.cn; 3 School of Agriculture and Biology, Shanghai Jiao Tong University, Shanghai 200240, China; 4 US Department of Agriculture, Western Regional Plant Introduction Station, Washington State University, Pullman, WA 99164, USA; jinguo.hu@usda.gov

**Keywords:** lettuce, germination, thermoinhibition, thermal stress, metabolomics

## Abstract

Temperature strongly influences lettuce (*Lactuca sativa* L.) seed germination. Different lettuce genotypes respond differently to higher temperatures or thermal stress. In this study, we evaluated the germination performance of 304 lettuce accessions incubated at three temperature settings, 21 °C, 28 °C and 35 °C, respectively, for 40 h. At 21 °C, seeds of all 304 accessions germinated with very well an average germination percentage of 87.72%; at 28 °C, the average germination percentage dropped to 42.84% and at 35 °C, the germination decreased to 1.01%. Then, we investigated changes in metabolic profiles of lettuce seed response to thermal stress using an untargeted metabolomics approach. Results suggested that seeds of thermal-sensitive and thermal-tolerant cultivars employed different metabolic strategies in response to thermal stress during germination. Thermal-sensitive buds accumulated more significant amounts of organic acids, amino acids, sugars, sterols, phenolic compounds and terpenoids compared to thermal-tolerant buds at 21 °C. Thermal-tolerant lettuce cultivar accumulated higher concentrations of amino acids, organic acids, sugars, sesquiterpene lactones, sterols, and fatty acids derivatives during the germination at 35 °C compared to germinated at 21 °C. This investigation paves the way to link the metabolomics to other external and internal factors affecting lettuce seed germination under thermal stress.

## 1. Introduction

Thermoinhibition refers to the phenomenon in which viable seeds failed to germinate at high temperature and it is recognized as an adaptive regeneration strategy for the cool-season crop to prevent seeds from germination at inappropriate high-temperature particularly in summer that may hamper the growth and development of the young plant [1]. In agricultural production, thermoinhibition limits the time and area for the production of various crops, including vegetables [2]. For example, most of the cultivars of the worldwide consumed vegetable crop, lettuce, exhibit seed thermoinhibition [3]. The optimal temperature for lettuce seed germination ranges from 18 to 21 °C [4]. The lettuce seed germination is greatly hampered by relatively high temperatures (>25 °C), such as in warm-season or in topical zones, when or where the temperature is always higher than the upper-temperature limitation of lettuce germination [2,3,5,6]. Therefore, understanding the mechanism of lettuce seed germination and thermoinhibition will greatly increase lettuce breeding efficiency in producing thermal tolerance cultivars to ensure good germination during warm season planting.

Due to its significance in both basic study and applied crop production, this fascinating phenomenon of thermoinhibition has been investigated for a long time. In the 1960s, researchers found that external factors such as light [7] and internal factors such as plant hormones [1,8] could alter seed germination under higher temperatures. ABA-related genes were more highly expressed when germination was inhibited, and GA- and ethylene-related genes were more highly expressed when germination was permitted [9]. In recent years, contemporary approaches, including genome sequencing, transcriptomics and proteomics have been deployed to gain insights of the complex nature of lettuce seed germination in response to heat stress [2,9,10]. A few key genes have been identified to be associated with the thermoinhibition of lettuce seeds. For instance, the upregulation of abscisic acid biosynthetic enzyme gene 9-cis-EPOXYCAROTENOID DIOXYGENASE4 results in germination thermoinhibition of lettuce seeds [3]. Considering that all thermoinhibition-affecting factors identified thus far will alter gene expression leading to synthesize or decompose certain metabolites, we intend to tackle this phenomenon from a different angle, using the metabolomic approach to gain information towards a better understanding of its mechanism.

Metabolomics has been widely employed to establish the connections between genotype and phenotype and is capable of identification and quantification of different classes of metabolites simultaneously and unbiasedly [11,12]. Non-targeted metabolomics approaches for lettuce have been developed and optimized for various research objectives previously. For instance, Garcia et al. using LC-MS based untargeted metabolomics approach compared differences of metabolome between two romaine lettuces after cutting and revealed that phenolic, terpenoid and lipid metabolites could explain the browning process in lettuce and be used as biomarkers to predict browning of fresh-cut lettuce [13,14]. An integrated GC-MS and LC-MS based untargeted metabolomic analysis was launched to compare the metabolic difference occurred in primary and secondary metabolism of lettuce hydroponically cultivated in organic and inorganic nitrogen culture, results suggested that organic nitrogen promoted the accumulation of glycosylated flavonoids, ascorbic acid and amino acids, but inhibited the content of phenolic acids and intermediates of the Krebs Cycle when compared to those lettuce cultivated under nitrate solution [15]. Yang et al. used combined GC×GC-TOF/MS and UPLC-IMS-QTOF/MS non-targeted metabolomic strategy to detect and relatively quantify metabolites in leafy and head lettuce cultivars, and found that 16 metabolites including phenolic acid derivatives, glycosylated flavonoids, and one iridoid were present at significantly different levels in leaf and head type lettuces [16]. However, to the best of our knowledge, using a large-scale untargeted metabolomics analysis to compare the metabolic diversity of lettuce seed germination and thermoinhibition has not been reported.

In this study, we evaluated the germination percentage of 274 lettuce accessions from Shanghai Agricultural Gene Center (SAGC) and 30 commercial cultivars purchased from the local market after being incubated for 40 h at 21 °C, 28 °C and 35 °C, respectively, in a germination chamber. Based on the obtained results, two lettuce accessions, one thermotolerant entry (‘s15k0106′, with the highest germination percentage at 35 °C) and one thermosensitive entry (‘s13k0062′, zero germination at 35 °C) were selected for further study using a metabolomics approach. We combined gas chromatography-mass spectrometry (GC/MS) and ultra-performance liquid chromatography-ion mobility spectrometry quadrupole time-of-flight mass spectrometry (UPLC-IMS-QTOF/MS) platforms to reveal the primary and secondary metabolites among the seed samples of the two selected accessions of dry seeds (seeds without germination tests), germinated seeds and ungerminated seeds at two temperature settings, 21 °C and 35 °C, respectively. We anticipate that this investigation will detect differences among the seed samples of different genotypes under different treatments and gain insight regarding the metabolic mechanism of lettuce seed thermoinhibition.

## 2. Results

### 2.1. Country of Origin, Seed Coat Color and Seed Weight of the Lettuce Germplasm Used in The Current Study

The 304 lettuce accessions used in this study were collected from the 34 countries (Supplemental Table S1). A large proportion (29.28%) of the germplasm was obtained from the Netherlands, followed by Turkey (17.76%), United States (13.49), China (11.51%) and another 28 countries (22.04%). For seed coat color, 194 accessions are white and 110 black. The average 100-grain weight for all 304 accessions was 0.118 g. Among all accessions, S14K0333 had the highest 100-grain weight of 0.183 g, and S15K050 had the lowest 100-grain weight of 0.066 g (Supplemental Table S1).

### 2.2. Evaluation of Seed Germination under Different Temperatures

**Figure 1:**
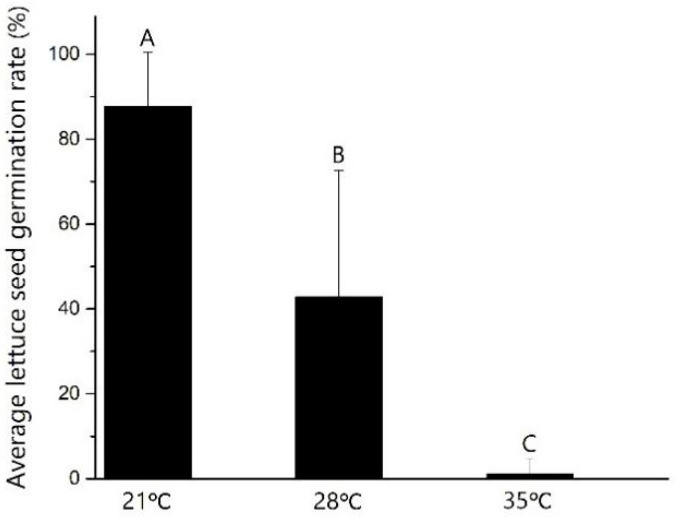
Average lettuce seed germination rate at different temperatures (%). The average lettuce seed germination rate of all 304 accessions was 87.72% at 21 °C, 42.84% at 28 °C and 1.01% at 35 °C, respectively. Different capital letters indicate significant differences, *p* < 0.01; LSD analysis (*n* = 304).

Lettuce genotypes differ greatly in their ability to germinate at different temperatures, as determined by the rates of germination. We observed a high average germination rate of 87.72% at 21 °C for all 304 accessions; however, the germination rate decreased to 42.84% at 28 °C and 1.01% at 35 °C, respectively; and these differences are statistically significant (*p* < 0.01), indicating lettuce seeds are sensitive to high-temperature stress (Figure 1). Sixteen accessions had high percentage germination of > 90 % at 28 °C, with s15k091 having the highest percentage germination (98.33%). Five accessions had a high percentage of germination (>10%) at 35 °C. s15k106 showed the highest percentage germination at 35 °C (39.67%), with corresponding germination percentages of 74.66% at 28 °C and 76.00% at 21 °C (Supplementary Table S1).

### 2.3. The Seed Color, Weight and Origin Region Influence on Germination Rates of Lettuce under Heat Stress

There was no relationship between dark and white seed coats on germination rates at 28 °C (46.43% vs. 51.12%, respectively; *p* = 0.243) or 35 °C (1.16% vs. 1.23%, respectively; *p* = 0.753). In addition, there were no relationships between 100-grain weight and seed color (0.12 g vs. 0.12 g for dark and white seeds, respectively; *p* = 0.267) or 100-grain weight and seed source (0.12 g vs. 0.11 g for accessions and cultivars, respectively; *p* = 0.157). However, there was a negative relationship between relative percent germination and 100-grain weight at 28 °C (R^2^ = −0.397, *p* < 0.01), but not at 35 °C.

### 2.4. Metabolic Profiles of Lettuce Seeds

Seeds of two cultivars, a thermosensitive variety (s13k0062) and a thermotolerant variety (s15k0106), were selected to investigate the metabolic mechanisms of seed germination at high temperature. A non-targeted metabolomics strategy combining GC/MS and UPLC-IMS-QTOF-MS was used to simultaneously detect and enable relative quantification of primary and secondary metabolites of lettuce [15]. Stable intensities were detected for the internal standards (L-2-chlorophenylalanine for GC/MS and EGCG for UPLC-IMS-QTOF/MS) in each sample. In addition, the drift retention times for the same internal standard peaks in different runs were less than 0.02 min in both GC/MS and UPLC-IMS-QTOF/MS analysis. Moreover, the Quality control (QC) samples clustered together in the center of the PCA scores in UPLC-IMS-QTOF/MS analysis (Supplemental Figure S1). Collectively, these results suggest the data acquisition was reproducible and robust. Overall, we extracted 998 features from the matrix in GC/MS analysis, and 8127 features were extracted from the UPLC-IMS-QTOF/MS.

### 2.5. Identification of Differential Metabolites

**Figure 2:**
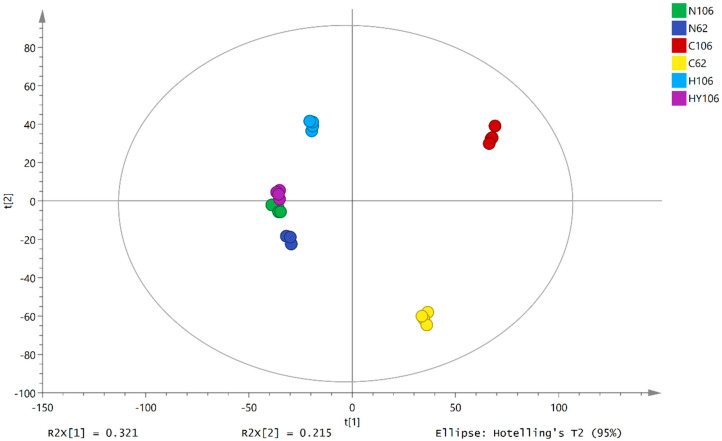
PCA score plots for different lettuce seed metabolomes. A five principle component-based PCA model (R^2^X _(cum)_ = 0.774, Q^2^_(cum)_ = 0.49) for GC/MS and UPLC-IMS-QTOF/MS data was obtained; the PCA analysis separated six different groups (N62: S13K0062 dry seeds; N106: S15K106 dry seeds; C106: buds of S15k0106 seeds germinated at 21 °C; C62: buds of S13k0062 seeds germinated at 21 °C; H106: S15K106 seeds fail to germination at 35 °C; HY106: buds of S15k0106 seeds germinated at 35 °C) and revealed differences in the relative content of metabolites present in different groups.

We performed unsupervised PCA analysis to determine the variations in metabolites between different cultivars and at different germination temperatures. A five-principle component-based robustness PCA model with a Q^2^_(cum)_ value of 0.491 was established. PC1 and PC2 explained 53.6% of the total variation, and clear separation was observed among the six different groups; the optimal germination temperature treatments (C106 and C62) were distributed far from the center of the coordinate axis (Figure 2). To identify potential variables, we screened the differential metabolites by comparing the fold change (> 2), *p*-value (< 0.01), and variable importance in projection (VIP) score (> 1.0) of the metabolites in each pair of comparisons and then identified these differential metabolites using online databases and our in-house database.

### 2.6. Metabolite Differences between Thermo-Tolerant and Thermo-Sensitive Seeds

**Figure 3:**
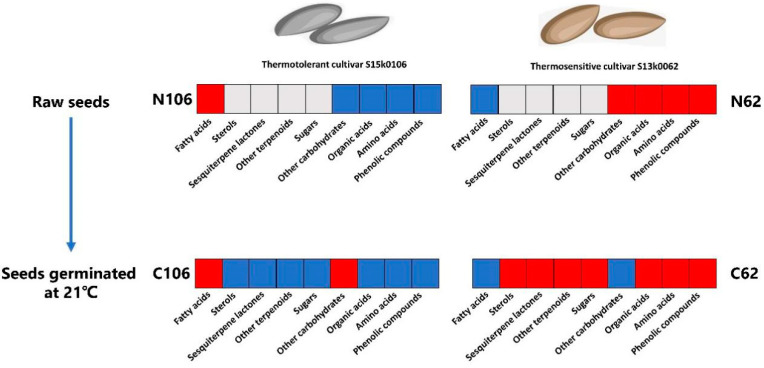
Differential metabolites detected between the thermotolerant and thermosensitive lettuce cultivars. Note: The heatmap indicates the differential metabolites extracted from raw S15k0106 (N106) and S13k0062 (N62) seeds and germinated buds of S15k0106 (C106) and S13k0062 (C62) seeds. A red box means that most of the metabolites in this class had higher relative abundances in a treatment than those in another in a pair of comparison (N106 vs. N62 or C106 vs. C62), while a blue box represents that relative abundances of most of the metabolites in this class were lower in a treatment than those in another between the two treatments; a gray box denotes no significance between two treatments.

A total of 867 metabolites were marked as differential metabolite candidates in the comparison between the thermotolerant seeds (N106) and thermosensitive seeds (N62); 11 of these metabolites were putatively annotated (Supplemental Table S2, Table S3, Table S4 and Figure S2A). N106 seeds had higher concentrations of the unsaturated long-chain fatty acids, including 9,12-octadecadienoic acid (Z, Z)-, 9-octadecenoic acid (E)- and oleic acid (Z)-, and saturated long-chain fatty acid (arachidic acid), than N62; N62 seeds had higher accumulation of some organic acids (lactic acid, quininic acid), amino acids (l-methionine, l-ornithine, l-asparagine, l-methionine), as well as arabinitol and caffeoyl-hexose isomer 2 (Table 1 and Figure 3).

We also compared the metabolic differences between buds of the thermotolerant seeds (C106) and thermosensitive seeds (C62) that were germinated at 21 °C, which led to the selection of 2175 candidate metabolites of which 39 were putatively identified (Supplemental Table S2, Table S3, Table S5 and Figure S2B). C106 had higher concentrations of fatty acid derivatives (arachidic acid, stearic acid, 9-octadecenoic acid, (E)-, palmitic acid, 1-monolinolein, lignoceric acid) and meso-erythritol than C62 (Table 2 and Figure 3). However, C62 accumulated higher levels of some organic acids (caffeic acid, pyruvic acid, glyceric acid, quininic acid, butanedioic acid, l-threonic acid, 4-hydroxybutanoic acid, malic acid), amino acids (4-aminobutanoic acid, phenylalanine, l-isoleucine, l-5-oxoproline, l-threonine, l-alanine, l-glutamic acid, l-valine, l-aspartic acid, l-proline, l-ornithine), sugars (glucose and fructose), oleic acid, (Z)-, sterols (campesterol, stigmasterol), terpenoids (α-tocopherol, lactucopicrin) and polyphenols (dihydroxybenzoic acid hexoside, dihydroxybenzoic acid, caffeoylquinic acid, caffeoylquinic acid hexoside, dihydrocaffeic acid hexose, caffeoyl-hexose isomer 1) than C106 (Table 2 and Figure 3). Overall, the thermosensitive cultivar s13k0062 tended to accumulate more significant amounts of phenolic compounds, amino acids, organic acids, sugars, terpenoids and sterols during germination.

### 2.7. Metabolite Differences between Thermotolerant Seeds Germinated at High Temperature and Optimal Temperature

**Figure 4:**
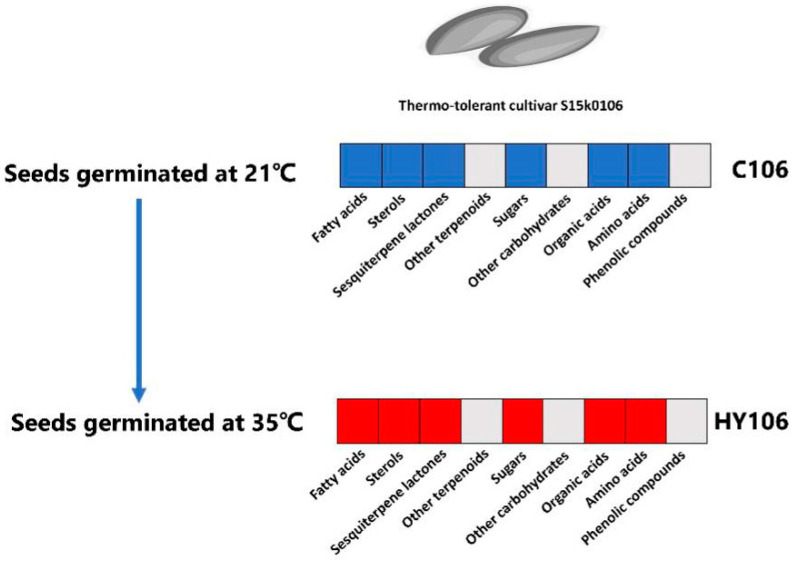
Differential metabolites detected between the thermotolerant lettuce seeds germinated at 21 °C and 35 °C. Note: The heatmap indicates the differential metabolites detected between buds of S15k0106 seeds germinated at 21 °C (C106) and 35 °C(HY106). A red box means that most of the metabolites in this class had higher relative abundances in HY106 compared to those in C106, while a blue box represents that relative abundances of most of the metabolites in this class were lower in C106 than those in HY106; a gray box denotes no significance between C106 and HY106.

For the comparison of C106 vs. HY106, a total number of 2445 of differential metabolites candidate, among them, 41 were putatively annotated (Supplemental Table S2, Table S3, Table S6, and Figure S2C). Compared to s15k0106 buds germinated at 21 °C (C106), we observed higher accumulation of organic acids, including tricarboxylic acid cycle (TCA) intermediates (butanedioic acid, fumaric acid, malic acid, citric acid), pyruvic acid, 4-hydroxybutanoic acid, quininic acid, glycolic acid, lactic acid, tartaric acid, glyceric acid, caffeic acid, amino acids (e.g., 4-aminobutanoic acid, phenylalanine, glycine, l-isoleucine, l-5-oxoproline, l-threonine, l-alanine, l-glutamic acid, l-valine, l-aspartic acid, l-proline, l-ornithine, l-methionine, l-leucine), sesquiterpene lactones (lactucopicrin), carbohydrates, including fructose, xylitol, arabinose, 1-(sn-Glycero-3-phospho)-1D-myo-inositol and lipid derivatives (including campesterol, stigmasterol, lignoceric acid, 9,12-octadecadienoic acid (Z, Z)-, palmitic acid, stearic acid, arachidic acid and 2-linoleoylglycerol) in s15k0106 seeds germinated at 35 °C (HY106). However, only some carbohydrates, including meso-erythritol and arabinitol, were observed to be lower in seeds germinated at 35 °C (HY106) than buds of the same cultivar at germinated at 21 °C (C106; Table 3 and Figure 4).

### 2.8. Metabolite Differences between Non-Germinated Seeds and Germinated Buds of Thermotolerant Cultivar at High Temperature

**Figure 5:**
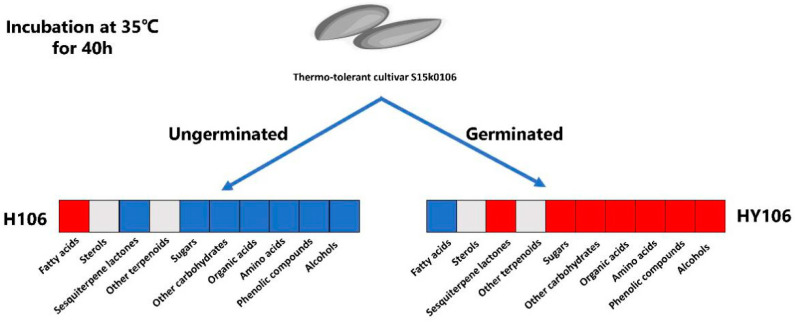
Differential metabolites detected between germinated buds and unterminated seeds of the thermal-tolerant lettuce cultivar. The heatmap indicates the differential metabolites between buds of S15k0106 germinated at 35 °C (HY106) and seeds of S15k0106 that did not germinate at 35 °C (H106). A red box means that most of the metabolites in this class had higher relative abundances in treatment compared to those in another, while a blue box represents that relative abundances of most of the metabolites in this class were lower in treatment than those in another; a gray box denotes no significance between H106 and HY106.

We assessed the metabolic differences between non-germinated seeds and germinated buds of the thermotolerant cultivar after incubation at 35 °C for 40 h (H106 vs. HY106). A total of 1220 differential metabolites candidates were detected, of which 39 candidates were putatively annotated (Supplemental Table S2, Table S3, Table S7 and Figure S2D). The non-germinated seeds had higher concentrations of uracil, fatty acids (arachidic acid, stearic acid, palmitic acid, 1-monolinolein) and R-3-hydroxybutyric acid than the germinated seeds. However, we detected higher concentrations of some organic acids (including butanedioic acid, quininic acid, glycolic acid, pyruvic acid, citric acid, ribonic acid, L-threonic acid, glyceric acid, fumaric acid, tartaric acid, 4-hydroxybutanoic acid, malic acid, propanedioic acid), alcohols (glycerol, 1-hexacosanol), carbohydrates (fructose, arabinitol, meso-erythritol, xylitol), amino acids (l-methionine, glycine, l-ornithine, l-alanine, phenylalanine, l-valine, l-aspartic acid, l-isoleucine, l-threonine, l-glutamic acid, l-proline, l-5-oxoproline), dihydrocaffeic acid hexose and 15-deoxylactucin-8-sulfate in germinated buds than the non-germinated seeds (Table 4 and Figure 5).

## 3. Discussion

### 3.1. Germination of Lettuce Germplasms is Inhibited by High Temperature

Lettuce seed germination is dramatically inhibited at high temperatures and will induce thermodormancy; the resulting poor seed germination and thermodormancy have detrimental effects on lettuce production in the field [17]. In this study, the average germination percentage of 304 lettuce accessions decreased significantly when the incubation temperature increased from 21 °C to 35 °C. These results are in line with previous studies, which suggested that lettuce seed germination was hampered at temperatures between 25 °C and 30 °C, depending on the genotype [9,18,19]. As the largest producer of lettuce worldwide, China contributes 56% of total world production [20]. In southern regions of China, the main area of lettuce production, over 30 days per year have a daily maximum temperature higher than 30 °C [21]. Lettuce genotypes differ greatly in their ability to germinate at high temperatures [4]. Therefore, there is an urgent need to identify lettuce cultivars that exhibit thermotolerance. In this study, three lettuce accessions showed great seed germination performance under thermal stress. the highest relative percent germination among the commercial cultivars was observed in S15K0154 at both 28 °C (90.61%) and 35 °C (19.86%); the relative percent germination of S15K0123 was 107 % at 28 °C, implying that heat stress (28 °C) had no effect or rather induced germination; it is worth mentioning that the entry s15k0106 (W6 29844, now designated as PI 667844) is a cultivar named ‘Florida Buttercrisp’ [22], which is from a warm area and, as its name implies, had the highest germination rate at 35 °C. These results suggest that the three accessions have strong potential use in the breeding of heat-resistant lettuce. Among the accessions, we observed that non-commercial cultivars had a lower average relative percent germination than the commercial cultivars at both 28 °C (47.85% vs. 62.03%) and 35 °C (1.13% vs. 1.58%). This result suggested that artificial selection plays an important role in improving germination rates in lettuce under heat stress.

Seed color had been previously reported to be associated with seed germination, dark-seeded lettuce is believed to have a higher percent of germination and increased seed vigor as well as lower pathogen susceptibility than white-seeded lettuce [23]. In this study, no relationship was observed between seed coats color and germination rate at different germination temperatures, suggesting that seed color is not a reliable predictor of final germination under heat stress conditions. The relationship between seed weight and vigor is controversial. In some crops (such as soybean and wheat), high seed weight has been linked to high seed vigor [24,25]. However, no relationship was observed in lettuce under cold conditions [26]. In this study, there was a negative relationship between relative percent germination and 100-grain weight at 28 °C (R^2^ = −0.397, *p* < 0.01), but not at 35 °C, suggesting that seed weight might be a useful predictor of germination at 28 °C. Further germination experiments at 21 °C to 35 °C are therefore required to explore higher correlation coefficients. There are several different opinions about the center of origin of cultivated lettuce. According to Lindqvist, it probably originated from Egypt [27]. According to Vavilov, cultivated lettuce originated in the Mediterranean area [28]. There was a single domestication event for cultivated lettuce that occurred ~10,800 years before present in the Kurdistan-Mesopotamia area [29,30]. In this study, the accessions from tropical countries, such as Vietnam, Kenya, and Laos, had a higher average relative percent germination at 28 °C (Supplementary Table S1). However, the germination assessment of lettuce germplasms from the world at different temperatures showed no obvious relationship between the germination of lettuce seed and the original seed region.

### 3.2. Metabolic Differences between Thermal-Sensitive and Thermal-Tolerant Cultivars during Germination at Proper Temperature

Seed germination is a complex physiological and metabolic process that involves different classes of metabolites, including amino acids, organic acids, available sugars, antioxidants and lipids [31,32,33]. In this study, the total abundances of organic acids, amino acids and carbohydrates were higher in thermosensitive cultivar s13k0062 during germination, when compared to thermotolerant cultivar s15k0106. The reason could be that seed germination processes increase degradation of lipids to fatty acids, proteins to free amino acids and starch to available sugars, and markedly increase the accumulation of organic acids [34,35]. Moreover, we observed that the buds of ‘s13k0062′ tended to accumulate more significant amounts of phenolic compounds and terpenoids compared to ‘S15k0106′during germination at 21 °C. In the early stage of germination, the seed must activate the antioxidant enzyme system to accumulate antioxidants (such as phenolic compounds and α-tocopherol) in order to scavenge excess reactive oxygen species and repair damage to the cell membrane [36]. The mevalonate pathway has been reported to be associated with lettuce seed germination. Isoprenoid biosynthesized via this pathway serves as a precursor for terpenoids (such as triterpenoids and sesquiterpenoids) and steroids [2]. These observations may explain the increased accumulation of terpenoids and phenolic compounds as antioxidants during germination in the seeds of the thermosensitive cultivar in this study.

### 3.3. Metabolite Requirements for Thermo-Tolerant Seeds Germination under Thermal Stress

Combined using GC/MS and UPLC-IMS-QTOF/MS platforms, we profiled the primary and secondary metabolites between the seed samples of thermotolerant lettuce cultivar of germinated at appropriate temperature (21 °C) and heat temperature (35 °C). We observed that thermotolerant lettuce cultivar accumulated higher concentrations of amino acids, organic acids, sugars, sesquiterpene lactones, sterols and fatty acids derivatives during the germination at 35 °C compared to germinated at 21 °C. Amino acids and some carbohydrates are recognized as osmotic substances; the higher accumulation in seeds under thermal stress may contribute to the stabilization of membranes and cellular osmotic pressure [37]. In addition, increased the contents of amino acids (such as glycine, l-alanine, proline, l-5-oxoproline, l-glutamic acid, and l-aspartic acid and l-ornithine) and sugars (e.g., fructose, xylitol and arabinose) function as compatible solute properties in Arabidopsis in response to temperature stress [37]. Aromatic amino acid (phenylalanine) is an important precursor of the phenylpropanoid pathway, increased level of L-phenylalanine might be the reason for the accumulation of phenolic compounds downstream. 4-aminobutanoic acid plays a crucial role in reactive oxygen species scavenging by regulating the gene expression of H_2_O_2_-producing genes (NADPH oxidase, peroxidase and amine oxidase) in the plant under stress [38]. Branched-chain amino acids, including valine, leucine and isoleucine as well as other amino acids that share the biosynthetic pathway, such as threonine and methionine function as compatible osmolytes under stress condition [39]. Additionally, these amino acids play as alternative electron donors for the mitochondrial electron transport chain under stress conditions [40].

Our results showed that pyruvic acid and TCA intermediates, such as butanedioic acid, fumaric acid, malic acid and citric acid, had higher accumulation in HY106 compared to C106. Pyruvic acid plays an essential precursor for the TCA cycle, and its accumulation results in the increased levels of TCA intermediates. In addition, the previous study reported that the activation of 4-aminobutanoic acid shunt resulted in TCA intermediates accumulation in Arabidopsis in response to dehydration due to the interplay between the 4-aminobutanoic acid shunt and the TCA cycle through various bypasses [41].

Notably, we observed that N106 seeds contain higher concentrations of the unsaturated long-chain fatty acids and saturated long-chain fatty acids than those in seeds of the thermosensitive cultivar. These results imply that heat-resistant of lettuce seed germination might be associated with lipids metabolism. Lipids are energy sources in seeds during embryo development, and the accumulation of high abundances of fatty acids is associated with energy metabolism in seeds [42]. Moreover, fatty acids and sterols are also involved in plant thermotolerant metabolism. Fatty acids are the major forms of the phospholipid bilayer, and their composition is crucial for maintaining membrane stability under stress [43]. Sterols act as regulators for the fluidity and permeability of cellular membranes [44]. In this study, we observed some steroids (stigmasterol and campesterol) accumulated at high levels in the seeds of the thermotolerant cultivar germinated at 35 °C (HY106). These findings are in line with previous observation in a perennial grass, hard fescue (*Festuca Trachyphylla*): ethyl sterols (fucosterol, stigmasterol, sitosterol, avenasterol) are positively associated with heat tolerance [45]. Fatty acid length in the stable membrane governs the thickness of lipid bilayers and tends to match the thickness of the membrane proteins [46]. To maintain membrane stability, s15k0106 seeds germinated at 35 °C (HY106) accumulated high concentration of long-chain fatty acid, such as lignoceric acid (C24), arachidic acid (C20), stearic acid (C18) and palmitic acid (C16). These results suggested that seeds of thermotolerant cultivar s15k0106 might employ different lipid metabolic strategies under thermal stress, including increased accumulation of long-chain fatty acids and sterols (stigmasterol and campesterol). These metabolic strategies could be beneficial for maintaining a proper lipid environment in the seed membrane for maintaining membrane stability and functionality during seeds adaptation to thermal stress [47].

Interestingly, we observed significantly higher accumulation of organic acids, amino acids, sesquiterpene lactones, lipids and carbohydrates in the buds of the thermotolerant accession s15k0106 when germinated at 35 °C (HY106) than when germinated at 21 °C (C106). A similar metabolic strategy was detected between s13k0062 buds germinated at 21 °C (C62) and s15k0106 buds germinated at 21 °C (C106). Twenty-three differential metabolites were common to both of these comparisons (HY106 vs. C106 and C106 vs. C62), suggesting that the thermotolerant cultivar s15k0106 may employ a similar metabolic strategy when germinated at high temperatures (35 °C) as the thermosensitive cultivar s13k0062 when germinated at 21 °C. While the thermosensitive s13k0062 seeds accumulated high levels of antioxidants (i.e., phenolic acids and α-tocopherol) during germination, and the accumulation of phenolic compounds were observed to be lower in thermotolerant s15k0106 seeds during germination at 35 °C compared to 21 °C. The reason could be that the thermotolerance of lettuce seeds undergoing germination is associated with the levels of reactive oxygen species in the seed. The levels of activated oxygen species were higher in the thermosensitive cultivar than the thermotolerant cultivar, which would lead to poorer heat resistance in the thermosensitive cultivar. Collectively, thermosensitive and thermotolerant cultivars employ distinctive metabolic strategies in response to heat stress. This work identified a large number of potential metabolic targets for further in-depth investigation of acquired seed tolerance to temperature stress. Moreover, these differential metabolites could be potential biomarkers for further screening of thermotolerant lettuce varieties for breeding projects.

## 4. Materials and Methods

### 4.1. Evaluate Seed Germination under Different Temperature

Used in the current study were 304 lettuce accessions, of which 257 were kindly offered by the Western Region Plant Introduction Station of the US Department of Agriculture, Pullman, WA, USA; 17 were provided by World Vegetable Centre, Taiwan, China; 30 commercial cultivars were purchased from the local market in Shanghai, China. Seeds were stored at −20 °C until starting the germination test.

For the germination test, three replicates of 100 seeds per accession or cultivar were placed in petri dishes (120 × 120 mm) on a single layer of Whatman #1 filter paper. A thin sponge was placed under the filter paper to retain moisture, and 4.5 mL of deionized water was added. The petri dishes were covered with lids to prevent evaporation then placed in incubators at 21 °C (proper temperature for lettuce seeds germination), 28 °C (upper limit temperature for lettuce seed) or 35 °C (upper limit temperature for thermotolerant cultivar seed) for 40 h in the dark to mimic the conditions of the soil at germination. Seed germination was recorded as the emergence of a radicle after incubation. Analysis of variance was performed on the germination data.

### 4.2. Two Accessions Are Selected for Metabolic Profiling

Two accessions, ‘s13k0062′ and ‘s15k0106′, were selected for metabolic profiling based on their germination performance under different temperatures. At 21 °C, both ‘s13k0062′ and ‘s15k0106′ germinated well with the germination percentages of 92.67% and 76.0%, respectively. However, at 28 °C, only less than 2% of the seeds of ‘s13k0062′ germinated, while the germination percentage of ‘s15k0106′ remained at 74.67%, almost the same as that at 21 °C. Still, at 35 °C, ‘s13k0062′ had 0% germination, while ‘s15k0106′ had 39.67% germination, the highest among all 304 accessions evaluated (Supplemental Table S1). Thus, we can say ‘s13k0062′ is thermosensitive and ‘s15k0106′ thermotolerant.

Four replicates of 100 seeds per cultivar were placed in an incubator to mimic seed germination using the method described above at 21 °C and 35 °C for 40 h. After incubation, all samples were collected and stored at −80 °C for metabolite extraction.

### 4.3. Metabolite Profiling

#### 4.3.1. GC-MS Analysis

A recently published extraction protocol [15] was followed for preparing samples for GC-MS analysis. Briefly, lettuce leaf sample (200 mg) was weighed and ground into fine powders in liquid nitrogen. Next, 1 mL ice-cold methanol: chloroform solution (3:1, v:v) was added. Samples were then vortexed at 6000 rpm for 15 s (repeated three times), and an internal standard (20 μL of 0.3 mg·mL^−1^ L-2-chlorophenylalanine) was added. After that, samples were centrifuged at 15,000× *g* for 10 min, and 300 μL supernatant of each sample was transferred to a new vial and vacuum-dried at room temperature. For derivatization, 80 μL of 20 mg·mL^−1^ methoxyamine hydrochloride (dissolved in pyridine) was added to the vial, and samples were then transferred into an oven and incubated at 37 °C for 1.5 h. Then, 80 μL of N, O-Bis(trimethylsilyl)trifluoroacetamide solution with 1% trimethylchlorosilane was added and followed by incubation at 70 °C for 1h. Supernatants of all samples were mixed, vacuum-dried and derivatized in the same manner to prepare QC samples. 1 μL derived extract of each sample was analyzed by using an Agilent 7890A-5975C GC/MS system (Agilent J&W Scientific, Folsom, CA, USA) coupled with a DB-5MS capillary column (30 m × 0.25 mm × 0.25 μm). The GC conditions were set as inlet temperature, 280 °C; After 6.5 min of solvent delay, the initial GC oven temperature was 60 °C; 1 min after injection, the GC oven temperature was raised to 300 °C at 5 °C min^−1^, and maintained at 300 °C for 11 min; transfer line temperature, 280 °C; ion source temperature, 230 °C; carrier gas (He) flow rate, 1mL min^−1^. MS condition: ion source, electron ionization; ionization energy, 70 eV; scan mode, full scan; scan range, *m/z* 33–600. The mixture of Supelco C7–C40 saturated alkanes standard ran prior to the samples to calculate the retention index of each feature. The QC samples were run at the start, middle, and end of the analysis.

#### 4.3.2. UPLC-IMS-QTOF/MS Analysis

The sample for UPLC-MS analysis was prepared and analyzed, as described previously [16]. In brief, lettuce seed samples were ground in liquid nitrogen into powder and then extracted with 1 mL methanol/water solution (80:20, *v/v*). Samples were then sonicated at 25 °C for 30 min and kept in the refrigerator (4 °C) for 12 h, followed by centrifugation at 12000× *g* for 10 min. 500 μL of the supernatant was collected for further analysis. QC samples were prepared by mixing the supernatant of all samples.

Samples (3 μL) were then analyzed by an Acquity class UPLC coupled with Vion IMS QTOF MS (Waters, Corp. Milford, MA, USA) using an Acquity UPLC HSS T3 column (100 mm × 2.1 mm, i, d.: 1.7 μm). Mobile phase: Water containing 0.1% formic acid (A) and acetonitrile containing 0.1% formic acid (B); flow rate: 0.4 mL min^−1^; UPLC conditions: gradient elution, 0–3 min, 0% B; 3–3.1 min, 0–5% B; 3.1–6 min, 5 to 20% B; 6–11 min, 20–50% B; 11–15 min 50 to 100% B, 15–17 min, 100% B, then initial conditions were restored for 5 min to equilibrate the column. For MS conditions, ion mode, negative; capillary voltage, 1.5 kV; source temperature, 115 °C; desolvation temperature, 500 °C; desolvation gas flow, 1000 L h^−1^; scan range, *m/z* 50–1000; collision energy, 20–40 eV. Data acquisition mode was performed in HDMS^E^ (data-independent acquisition type in ion-mobility). A 5 μL of 0.2 mg mL^−1^ EGCG was added as an internal standard. QC samples were inserted into each sample branch.

### 4.4. Data Pre-Processing and Normalization

Raw data acquired from GC/MS and UPLC-IMS-QTOF/MS were pre-processed by LECO Chroma TOF and Waters Progenesis QI, respectively. The raw data was performed in the pre-process procedures, including peak picking, baselining and alignment. Then, the pre-processing data matrix containing feature name (named as retention time and *m/z*,), sample information (four biological replicates per sample), relative abundance (calculated by peak area) was prepared and submitted to MetaboAnalyst (https://www.metaboanalyst.ca/). After that, the data matrix was performed three categories of normalization, including normalization by median, log transformation and auto-scaling via online data analysis software MetaboAnalyst embedded algorithm.

### 4.5. Data Analysis

#### 4.5.1. Study Design

**Figure 6:**
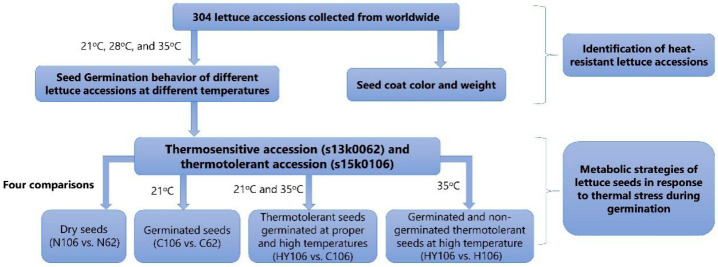
The workflow of the current study.

Four comparisons between different groups were set to investigate the difference in metabolomes between the two accessions of 1. dry seeds (N106 vs. N62, denoting ‘s15k0106′of and ‘s13k0062′, respectively); 2. the germinated seeds of the two accessions at 21 °C (C106 vs. C62); 3. the germinated seeds of ‘s15k0106′ at 21 °C and 35 °C (HY106 vs. C106); and 4) the seeds of ‘s15k0106′ germinated and non-germinated at 35 °C (HY106 vs. H106). The study design is shown in Figure 6.

#### 4.5.2. Univariate and Multivariate Statistical Analysis

The principal component analysis (PCA) of all samples and QCs was performed in SIMCA-P software (version 14.1, Sartorius Stedim Biotech, Umeå, Sweden). To identify potential variables that contribute to differentiation of four pairs of comparison, fold change and student’s t-test value were calculated, and VIP value was calculated by conducting the partial least squares-discriminant analysis (PLS-DA). All these parameters were obtained by the embedded algorithm in MetaboAnalyst.

#### 4.5.3. Differential Metabolites Screening

The differential metabolites were screened by parameters including fold change > 2, VIP value > 1, and *p*-value < 0.01. Then, these ‘candidates’ were marked for compound identification to explore their biological roles during seed germination.

#### 4.5.4. Compound Identification

Candidates from GC-MS were identified through compared with reference and online database including our in-house lettuce metabolites library [15], NIST 2014 (Similarity > 800) and Golm Metabolome Database (CV of Retention Index < 1.5%); candidates from UPLC-IMS-QTOF/MS metabolites were identified through compared with our in-house database [16], which included mass to charge, retention time, MS^2^ fragments. All matching results were then supervised manually.

## 5. Conclusions

In conclusion, the germination of lettuce germplasm reduces as temperature increases. Artificial selection has improved the germination rates of lettuce under heat stress. Our comparative metabolomic analysis revealed that the accumulation of organic acids, amino acids, terpenoids, phenolic compounds, carbohydrates and lipids are involved in lettuce seed germination and thermoinhibition. Thermosensitive seeds accumulated more significant amounts of organic acids, amino acids, sugars, sterols, phenolic compounds and terpenoids compared to thermotolerant seeds at 21 °C. Thermotolerant lettuce cultivar accumulated higher concentrations of amino acids, organic acids, sugars, sesquiterpene lactones, sterols and fatty acids derivatives during the germination at 35 °C compared to germinated at 21 °C. Overall, our findings indicate the complexity of lettuce germination under abiotic stress conditions. Seeds of thermal-sensitive and thermal-tolerant cultivars employed different metabolic strategies in response to thermal stress during germination. Moreover, heat-resistant accessions were identified for future breeding programs that aim to develop new cultivars suitable for extended summer production in temperate or tropical climates when soil temperatures are higher than the optimal germination temperature. Further studies are now required to assess the ability of these accessions to germinate rapidly at higher temperatures under field conditions.

## Figures and Tables

**Table 1:** Differential metabolites extracted from S15k0106 (N106) seeds and S13k0062 (N62) seeds.

No.	Compound	Log2 (FC N106/N62)	*p*-Value	VIP Value
GC_95	Lactic acid	−1.90	1.70 × 10^−5^	1.26
GC_361	L-Methionine	−1.12	1.70 × 10^−5^	1.26
GC_453	Asparagine	−1.75	3.58 × 10^−3^	1.17
GC_484	Arabinitol	−1.03	3.31 × 10^−5^	1.25
GC_537	L-Ornithine	−1.19	1.73 × 10^−5^	1.26
GC_554	Quininic acid	−1.01	9.85 × 10^−5^	1.25
GC_686	9,12-Octadecadienoic acid (Z, Z)-	1.00	2.69 × 10^−4^	1.23
GC_689	9-Octadecenoic acid, (E)-	1.56	4.09 × 10^−5^	1.25
GC_692	Oleic acid, (Z)-	1.11	1.00 × 10^−3^	1.21
GC_786	Arachidic acid	1.06	9.46 × 10^−4^	1.21
LC_4594	Caffeoyl-hexose 2	−1.55	1.55 × 10^−5^	1.26

Note: Fold change of different metabolites between N106 and N62 is reported as Log2(FC N106/N62).

**Table 2:** Differential metabolites extracted from germinated buds of S15k0106 (C106) and S13k0062 (C62) seeds.

No.	Compound	Log2 (FC C106/C62)	*p*-Value	VIP Value
GC_89	Pyruvic acid	−1.83	4.86 × 10^−5^	1.12
GC_115	l-Alanine	−2.13	5.94 × 10^−4^	1.09
GC_185	l-Valine	−2.34	4.27 × 10^−4^	1.09
GC_200	4-Hydroxybutanoic acid	−3.46	8.85 × 10^−5^	1.11
GC_233	l-Isoleucine	−1.83	2.10 × 10^−5^	1.13
GC_236	l-Proline	−5.33	6.79 × 10^−5^	1.12
GC_249	Butanedioic acid	−2.69	5.53 × 10^−8^	1.14
GC_258	Glyceric acid	−1.85	2.17 × 10^−5^	1.13
GC_290	l-Threonine	−2.01	2.15 × 10^−8^	1.14
GC_349	Malic acid	−6.45	1.42 × 10^−9^	1.14
GC_359	meso-Erythritol	1.56	9.35 × 10^−6^	1.13
GC_362	l-5-Oxoproline	−1.92	4.36 × 10^−7^	1.14
GC_364	l-Aspartic acid	−2.40	4.96 × 10^−7^	1.14
GC_368	4-Aminobutanoic acid	−1.01	2.27 × 10^−4^	1.10
GC_391	l-Threonic acid	−2.19	7.27 × 10^−7^	1.14
GC_425	l-Ornithine	−5.37	1.53 × 10^−4^	1.11
GC_427	l-Glutamic acid	−2.28	1.70 × 10^−6^	1.14
GC_430	Phenylalanine	−1.27	4.23 × 10^−8^	1.14
GC_554	Quininic acid	−1.88	1.27 × 10^−6^	1.14
GC_557	Fructose	−2.91	6.58 × 10^−6^	1.13
GC_569	Glucose	−1.67	3.44 × 10^−5^	1.12
GC_606	Caffeic acid	−1.25	4.12 × 10^−6^	1.13
GC_627	Palmitic acid	2.91	7.42 × 10^−8^	1.14
GC_689	9-Octadecenoic acid, (E)-	3.21	5.83 × 10^−6^	1.13
GC_692	Oleic acid, (Z)-	−44.26	5.41 × 10^−15^	1.14
GC_702	Stearic acid	3.77	1.55 × 10^−8^	1.14
GC_786	Arachidic acid	3.84	1.33 × 10^−9^	1.14
GC_874	1-Monolinolein	1.43	2.11 × 10^−7^	1.14
GC_897	Lignoceric acid	1.17	1.18 × 10^−4^	1.11
GC_948	α-Tocopherol	−1.56	1.19 × 10^−4^	1.11
GC_965	Campesterol	−1.23	8.03 × 10^−5^	1.12
GC_968	Stigmasterol	−1.35	6.34 × 10^−5^	1.12
LC_443	Caffeoylquinic acid	−2.65	2.06 × 10^−4^	1.10
LC_1072	Dihydrocaffeic acid hexose	−3.52	6.67 × 10^−10^	1.14
LC_1096	Dihydroxybenzoic acid	−2.25	1.85 × 10^−7^	1.14
LC_1152	Caffeoyl-hexose 1	−3.97	7.57 × 10^−9^	1.14
LC_1736	Lactucopicrin	−2.28	8.49 × 10^−6^	1.13
LC_1777	Dihydroxybenzoic acid hexoside	−2.01	1.65 × 10^−7^	1.14
LC_4473	Caffeoylquinic acid hexoside	−3.15	9.40 × 10^−8^	1.14

Note: Fold change of different metabolites between C106 and C62 is reported as Log2(FC C106/C62).

**Table 3:** Differential metabolites detected between buds of S15k0106 seeds germinated at 35 °C (HY106) and 21 °C (C106).

No.	Compound	Log2 (FC HY106/C106)	*p*-Value	VIP Value
GC_89	Pyruvic acid	8.18	1.16 × 10^−7^	1.13
GC_95	Lactic acid	5.11	5.76 × 10^−8^	1.13
GC_103	Glycolic acid	5.10	3.68 × 10^−7^	1.13
GC_115	l-Alanine	7.60	8.95 × 10^−7^	1.12
GC_122	Glycine	6.06	9.84 × 10^−6^	1.12
GC_185	l-Valine	7.74	8.89 × 10^−7^	1.12
GC_200	4-Hydroxybutanoic acid	9.13	2.47 × 10^−7^	1.13
GC_221	l-Leucine	8.36	1.41 × 10^−6^	1.12
GC_233	l-Isoleucine	6.80	8.26 × 10^−8^	1.13
GC_236	l-Proline	8.13	5.74 × 10^−6^	1.12
GC_249	Butanedioic acid	6.81	1.79 × 10^−8^	1.13
GC_258	Glyceric acid	7.88	3.10 × 10^−8^	1.13
GC_270	Fumaric acid	9.83	2.10 × 10^−8^	1.13
GC_290	l-Threonine	7.59	1.23 × 10^−8^	1.13
GC_349	Malic acid	12.77	9.02 × 10^−10^	1.13
GC_359	meso-Erythritol	-2.49	5.77 × 10^−6^	1.12
GC_361	l-Methionine	6.21	6.76 × 10^−8^	1.13
GC_362	l-5-Oxoproline	7.61	3.01 × 10^−8^	1.13
GC_364	l-Aspartic acid	8.73	2.22 × 10^−8^	1.13
GC_368	4-Aminobutanoic acid	6.54	5.38 × 10^−6^	1.12
GC_425	l-Ornithine	11.77	2.35 × 10^−6^	1.12
GC_427	l-Glutamic acid	8.47	5.08 × 10^−8^	1.13
GC_430	Phenylalanine	6.41	2.81 × 10^−8^	1.13
GC_439	Tartaric acid	8.50	2.50 × 10^−8^	1.13
GC_457	Arabinose	4.81	3.56 × 10^−6^	1.12
GC_468	Xylitol	4.18	2.86 × 10^−7^	1.13
GC_484	Arabinitol	-2.81	5.47 × 10^−6^	1.12
GC_538	Citric acid	6.14	1.22 × 10^−7^	1.13
GC_554	Quininic acid	4.25	4.31 × 10^−7^	1.13
GC_557	Fructose	8.55	1.27 × 10^−6^	1.12
GC_606	Caffeic acid	3.72	6.53 × 10^−4^	1.07
GC_627	Palmitic acid	1.81	4.62 × 10^−5^	1.10
GC_686	9,12-Octadecadienoic acid (Z, Z)-	4.60	3.84 × 10^−7^	1.13
GC_702	Stearic acid	1.56	7.71 × 10^−5^	1.10
GC_786	Arachidic acid	1.60	1.01 × 10^−4^	1.10
GC_863	2-Linoleoylglycerol	4.88	7.03 × 10^−7^	1.12
GC_897	Lignoceric acid	4.64	3.60 × 10^−7^	1.13
GC_965	Campesterol	6.54	1.48 × 10^−7^	1.13
GC_968	Stigmasterol	6.98	1.29 × 10^−7^	1.13
LC_1736	Lactucopicrin	5.17	5.46 × 10^−7^	1.12
LC_4802	1-(sn-Glycero-3-phospho)-1D-myo-inositol	6.97	2.13 × 10^−4^	1.09

Note: Fold change of different metabolites between HY106 and C106 is reported as Log2(FC HY106/C106).

**Table 4:** Differential metabolites between buds of S15k0106 germinated at 35 °C (HY106) and seeds of S15k0106 that did not germinate at 35 °C (H106).

No.	Compound	Log2 (FC H106/HY106)	*p*-Value	VIP Value
GC_103	Glycolic acid	−2.29	2.12 × 10^−5^	1.23
GC_108	Pyruvic acid	−2.40	1.56 × 10^−5^	1.23
GC_115	L-Alanine	−3.14	2.64 × 10^−5^	1.23
GC_122	Glycine	−2.68	1.02 × 10^−4^	1.22
GC_146	R-3-Hydroxybutyric acid	1.78	5.14 × 10^−3^	1.12
GC_177	Propanedioic acid	−34.26	5.88 × 10^−12^	1.24
GC_185	L-Valine	−3.42	2.20 × 10^−5^	1.23
GC_200	4-Hydroxybutanoic acid	−5.27	4.09 × 10^−3^	1.13
GC_223	Glycerol	−1.07	3.67 × 10^−3^	1.14
GC_233	L-Isoleucine	−3.66	1.03 × 10^−5^	1.23
GC_236	L-Proline	−4.53	2.99 × 10^−7^	1.24
GC_249	Butanedioic acid	−1.14	4.45 × 10^−3^	1.13
GC_258	Glyceric acid	−3.90	1.87 × 10^−6^	1.24
GC_261	Uracil	2.41	2.39 × 10^−3^	1.15
GC_270	Fumaric acid	−4.54	1.01 × 10^−5^	1.23
GC_290	L-Threonine	−3.72	5.43 × 10^−6^	1.24
GC_349	Malic acid	−6.99	1.50 × 10^−8^	1.24
GC_359	meso-Erythritol	−1.73	4.82 × 10^−4^	1.19
GC_361	L-Methionine	−2.00	6.25 × 10^−4^	1.19
GC_362	L-5-Oxoproline	−4.81	1.07 × 10^−7^	1.24
GC_364	L-Aspartic acid	−3.44	8.99 × 10^−6^	1.23
GC_391	L-Threonic acid	−3.20	1.49 × 10^−5^	1.23
GC_425	L-Ornithine	−2.72	2.15 × 10^−6^	1.24
GC_427	L-Glutamic acid	−3.80	3.32 × 10^−6^	1.24
GC_430	Phenylalanine	−3.35	7.81 × 10^−6^	1.23
GC_439	Tartaric acid	−5.25	6.12 × 10^−8^	1.24
GC_468	Xylitol	−2.46	9.96 × 10^−5^	1.22
GC_484	Arabinitol	−1.37	1.75 × 10^−4^	1.21
GC_513	Ribonic acid	−2.90	4.47 × 10^−5^	1.22
GC_538	Citric acid	−2.77	1.19 × 10^−4^	1.22
GC_554	Quininic acid	−1.95	3.91 × 10^−5^	1.23
GC_557	Fructose	−1.80	8.22 × 10^−3^	1.10
GC_627	Palmitic acid	1.68	6.45 × 10^−5^	1.22
GC_702	Stearic acid	1.94	3.32 × 10^−5^	1.23
GC_786	Arachidic acid	2.00	2.69 × 10^−5^	1.23
GC_874	1-Monolinolein	1.54	1.22 × 10^−4^	1.21
GC_917	1-Hexacosanol	−2.44	4.29 × 10^−6^	1.24
LC_1072	Dihydrocaffeic acid hexose	−1.69	7.35 × 10^−5^	1.22
LC_1239	15-deoxylactucin-8-sulfate	−4.41	3.54 × 10^−7^	1.24

Note: Fold change of different metabolites between H106 and HY106 is reported as Log2(FC H106/HY106).

## Supplementary Materials

Supplementary materials can be found at https://www.mdpi.com/1422-0067/21/4/1481/s1.

## Abbreviations

Quality control	QC
Mass spectrometry	MS
Shanghai Agricultural Gene Center	SAGC
Gas chromatography-mass spectrometry	GC/MS
Ultra-performance liquid chromatography-ion mobility spectrometry quadrupole time-of-flight mass spectrometry	UPLC-IMS-QTOF/MS
Principal component analysis	PCA
Partial least squares-discriminant analysis	PLS-DA
Tricarboxylic acid cycle	TCA
Variable importance in projection	VIP
