# Supplementary material for: Distinct Metabolome Changes during Seed Germination of Lettuce (Lactuca sativa L.) in Response to Thermal Stress as Revealed by Untargeted Metabolomics Analysis
Source: Int J Mol Sci. 2020 Feb 21;21(4):1481. doi: 10.3390/ijms21041481 (PMC7073097; doi:10.3390/ijms21041481)
Supplement: Supplementary file 1 [file ijms-21-01481-s001.zip › Supplemental figures.docx]

**Supplemental Figure Captions**

**Supplemental Figure S1.** PCA scores of the quality controls (QC) and samples in UPLC-IMS-QTOF/MS analysis. QC samples clustered together in the center of the PCA scores in UPLC-IMS-QTOF/MS analysis.

**Supplemental Figure S2.** PLS-DA score scatter plot of the four pair of comparisons. Note: A, N62 vs. N106 (3 compounds based model, R^2^ = 0.99, Q^2^ = 0.97, and Accuracy = 1.0); B, C62 vs. C106(2 compounds based model, R^2^ = 0.99, Q^2^ = 0.99, and Accuracy = 1.0); C, C106 vs. HY 106(3 compounds based model, R^2^ = 0.99, Q^2^ = 0.99, and Accuracy = 1.0); D, H106 vs. HY106(3 compounds based model, R^2^ = 1, Q^2^ = 0.97, and Accuracy = 1.0).

**
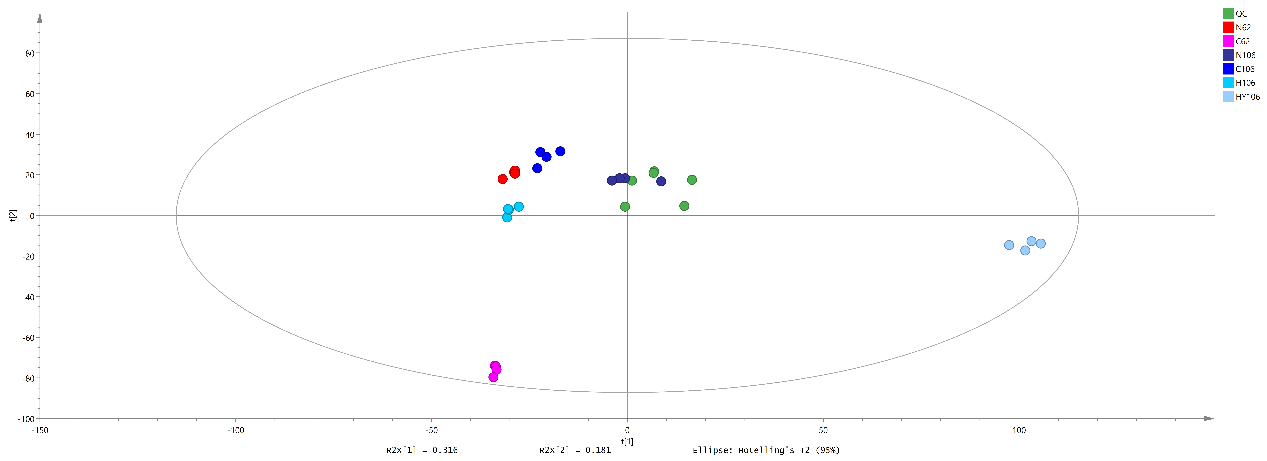
**

**Supplemental Figure S1.** PCA scores of the quality controls (QC) and samples in UPLC-IMS-QTOF/MS analysis. QC samples clustered together in the center of the PCA scores in UPLC-IMS-QTOF/MS analysis.


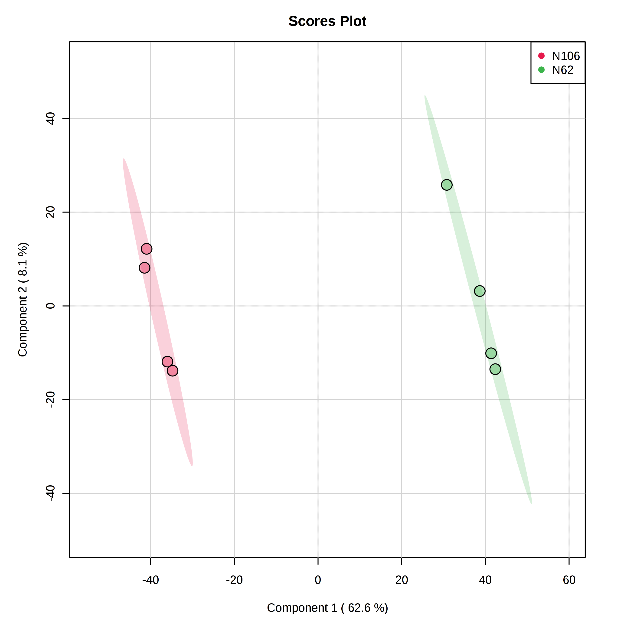

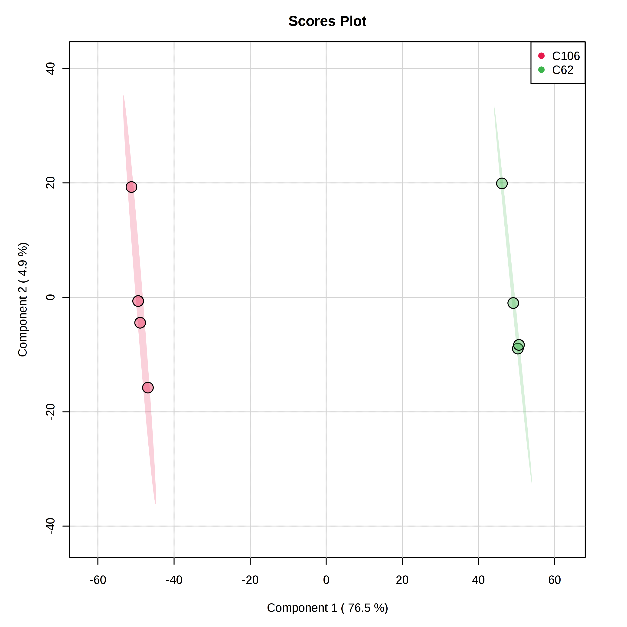

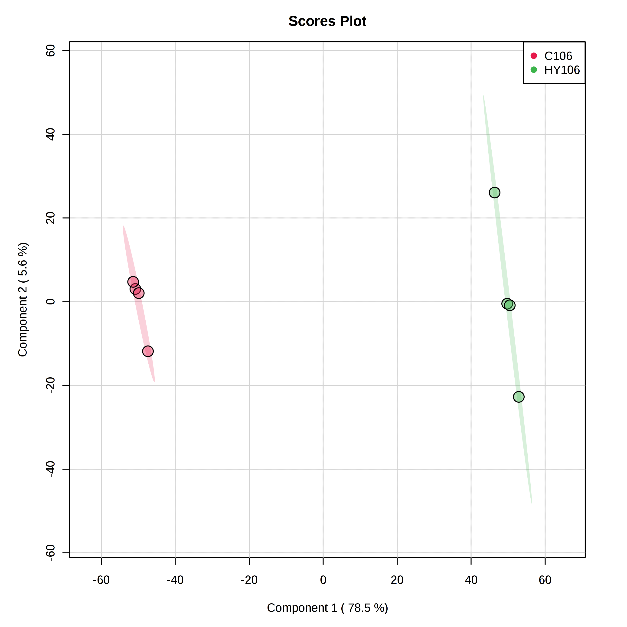

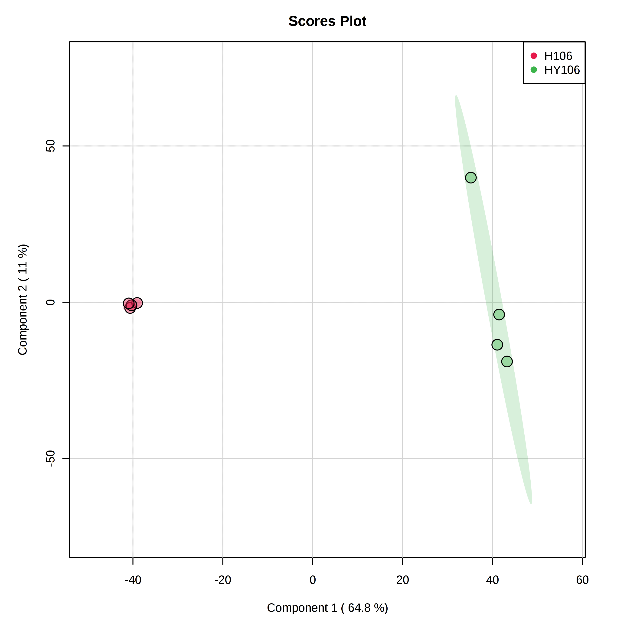


**B**

**A**

**D**

**C**

**Supplemental Figure S2.** PLS-DA score scatter plot of the four pair of comparisons. Note: A, N62 vs. N106 (3 compounds based model, R^2^ = 0.99, Q^2^ = 0.97, and Accuracy = 1.0); B, C62 vs. C106(2 compounds based model, R^2^ = 0.99, Q^2^ = 0.99, and Accuracy = 1.0); C, C106 vs. HY 106(3 compounds based model, R^2^ = 0.99, Q^2^ = 0.99, and Accuracy = 1.0); D, H106 vs. HY106(3 compounds based model, R^2^ = 1, Q^2^ = 0.97, and Accuracy = 1.0).
